# Kynurenic Acid: A Novel Player in Cardioprotection against Myocardial Ischemia/Reperfusion Injuries

**DOI:** 10.3390/ph16101381

**Published:** 2023-09-28

**Authors:** Rima Kamel, Delphine Baetz, Naïg Gueguen, Lucie Lebeau, Agnès Barbelivien, Anne-Laure Guihot, Louwana Allawa, Jean Gallet, Justine Beaumont, Michel Ovize, Daniel Henrion, Pascal Reynier, Delphine Mirebeau-Prunier, Fabrice Prunier, Sophie Tamareille

**Affiliations:** 1 MITOVASC, SFR ICAT, CNRS 6015, INSERM U1083, Université d’Angers, F-49000 Angers, France; rimakamel@hotmail.com (R.K.); naig.gueguen@univ-angers.fr (N.G.); lucie.lebeau@univ-angers.fr (L.L.); anne-laure.guihot@univ-angers.fr (A.-L.G.); louwana_allawa@hotmail.fr (L.A.); daniel.henrion@univ-angers.fr (D.H.); pareynier@chu-angers.fr (P.R.); deprunier@chu-angers.fr (D.M.-P.); faprunier@chu-angers.fr (F.P.); 2 Laboratoire CarMeN, INSERM U1060, INRA U1397, Université Claude Bernard Lyon 1, F-69500 Bron, France; delphine.baetz@univ-lyon1.fr (D.B.); michel.ovize@gmail.com (M.O.); 3 Service de Biochimie et Biologie Moléculaire, CHU Angers, F-49000 Angers, France; 4 Service de Cardiologie, CHU Angers, F-49000 Angers, France; jgallet@chu-angers.fr; 5 Service d’Explorations Fonctionnelles Cardiovasculaires & CIC de Lyon, Hôpital Louis Pradel, Hospices Civils de Lyon, F-69000 Lyon, France

**Keywords:** kynurenic acid, myocardial infarction, cardioprotection, mitophagy, oxidative stress, kynurenine pathway, ischemia/reperfusion injuries, cell death, hypoxia/reoxygenation

## Abstract

Background: Myocardial infarction is one of the leading causes of mortality worldwide; hence, there is an urgent need to discover novel cardioprotective strategies. Kynurenic acid (KYNA), a metabolite of the kynurenine pathway, has been previously reported to have cardioprotective effects. However, the mechanisms by which KYNA may be protective are still unclear. The current study addressed this issue by investigating KYNA’s cardioprotective effect in the context of myocardial ischemia/reperfusion. Methods: H9C2 cells and rats were exposed to hypoxia/reoxygenation or myocardial infarction, respectively, in the presence or absence of KYNA. In vitro, cell death was quantified using flow cytometry analysis of propidium iodide staining. In vivo, TTC-Evans Blue staining was performed to evaluate infarct size. Mitochondrial respiratory chain complex activities were measured using spectrophotometry. Protein expression was evaluated by Western blot, and mRNA levels by RT-qPCR. Results: KYNA treatment significantly reduced H9C2-relative cell death as well as infarct size. KYNA did not exhibit any effect on the mitochondrial respiratory chain complex activity. SOD2 mRNA levels were increased by KYNA. A decrease in p62 protein levels together with a trend of increase in PARK2 may mark a stimulation of mitophagy. Additionally, ERK1/2, Akt, and FOXO3α phosphorylation levels were significantly reduced after the KYNA treatment. Altogether, KYNA significantly reduced myocardial ischemia/reperfusion injuries in both in vitro and in vivo models. Conclusion: Here we show that KYNA-mediated cardioprotection was associated with enhanced mitophagy and antioxidant defense. A deeper understanding of KYNA’s cardioprotective mechanisms is necessary to identify promising novel therapeutic targets and their translation into the clinical arena.

## 1. Introduction

Myocardial infarction (MI) remains one of the leading causes of morbidity and mortality in developed countries [1]. MI is a complex and a serious clinical problem that causes irreversible damage to the heart. Timely reperfusion, either by thrombolytic therapy or by angioplasty, is a prerequisite to the salvaging of ischemic myocardium and improvement of patient’s prognosis. However, myocardial reperfusion is paradoxically associated with more damage to the heart including irreversible death of cardiomyocytes. Four types of myocardial ischemia/reperfusion injuries have been described including myocardial stunning, the no-reflow phenomenon, reperfusion arrhythmia, and lethal reperfusion injury [2]. Whilst several pharmacological and ischemic conditioning strategies were proven capable of reducing myocardial reperfusion injuries in animal models and clinical studies, no effective cardioprotective strategy has been translated into the clinical arena [3,4]. Therefore, innovative cardioprotective therapies are urgently required to prevent myocardial I/R injuries and improve clinical outcomes.

The kynurenine pathway (KP) is the major route of tryptophan degradation [5,6]. Approximately 95% of free tryptophan is oxidized into kynurenine, a rate-limiting step controlled by indolamine-2,3-dioxygenase (IDO) or tryptophan-2,3-dioxygenase (TDO) in the liver. Thereafter, kynurenine is metabolized into either kynurenic acid (KYNA), or quinolinic acid, via reactions catalyzed by kynurenine aminotransferase (KAT) and kynurenine 3-monooxygenase (KMO), respectively. KP metabolism is altered in various diseases such as immune-related disorders, endocrine and metabolic conditions, cancers, and neuropsychiatric diseases [7,8]. It has also been involved in chronic pain such as migraine and neuropathic pain [9]. Thus, KP metabolites have been described as bioactive compounds and carry out a broad range of biological functions, such as oxidant, antioxidant, anti-inflammatory, neurotoxic, neuroprotective, and/or immunomodulatory activities [6,10,11]. Interestingly, their effects depend on their local concentration and cellular environment, metabolic activity as well as complex positive and negative feedback loops [12]. Recently, a study reported that downregulation of KP might improve the outcome of acute mesenteric ischemia in rats [13]. Moreover, plasma levels or ratios of KP metabolites were associated with adverse clinical outcomes [14].

*Recently, alteration of KP* has also been implicated in the pathophysiology of cardiovascular diseases (CVD) [15,16]. Several CVD are associated with the overactivation of kynurenine pathway, including hypertension, atherosclerosis, ischemic heart diseases, and stroke. Interestingly, KP metabolites have been described as potential diagnostic and prognostic biomarkers in cardiovascular diseases [17]. Kynurenine or its metabolites supplementation have been shown to improve the outcome of stroke [17]. In serum of CVD patients, there were increases in kynurenine, kynurenine/Trp ratio (KTR), quinolinic acid, KYNA, and 5-hydroxyindoleacetic acid, whereas in patients presenting documented peripheral atherosclerosis, and ischemic heart diseases decreases in concentrations of Trp and serotonin were observed [18]. Another study found that KTR levels predict acute coronary events in older adults without previous coronary heart disease [19]. Moreover, high levels of plasma kynurenines predicted increased risk of acute myocardial infarction in patients with suspected stable angina pectoris. Altogether, these studies give evidence that the kynurenine pathway metabolites may play a role in heart injuries. Nevertheless, the role of KP in the heart is not completely clear yet. In previous works, we showed a plasmatic kynurenine concentration increase in rats and humans following the cardioprotective strategy of remote ischemic conditioning (RIC), suggesting a link between the kynurenine pathway and cardioprotection [20,21,22]. Interestingly, RIC-induced cardioprotection was lost in rats that received 1-methyl-tryptophan (1-MT) pretreatment, an inhibitor of kynurenine synthesis from TRP [21]. Moreover, rats receiving a kynurenine intraperitoneal injection 10 min before a myocardial ischemia/reperfusion exhibited a smaller infarct size compared to the vehicle-treated rats [20]. 

KYNA is a key metabolite of the kynurenine-tryptophan pathway with pleiotropic effects [23]. KYNA has anti-inflammatory and immunosuppressive functions as well as antioxidant and neuroprotective properties [23,24]. Importantly, in 2016, Olenchock et al. reported KYNA’s role in cardioprotection using ex vivo and in vivo myocardial ischemia/reperfusion mouse models [25]. A more recent study showed that KYNA may play a significant role in mediating cardiac protection following acute kidney injury [26]. These findings reinforce a potential major role of KYNA in protecting the heart against ischemia/reperfusion injuries. However, the mechanism by which KYNA is cardioprotective was not unraveled and thus remains largely unknown. Thus, the objective of the present study was to investigate KYNA’s cardioprotective effect and to elucidate potential cardioprotective pathways using both an in vivo rat myocardial infarction model and an in vitro hypoxia/reoxygenation H9C2 cell model.

## 2. Results

### 2.1. KYNA Reduced In Vitro Cell Death and Prevented Mitochondrial Membrane Potential Decrease after Hypoxia/Reoxygenation

**Figure 1:**
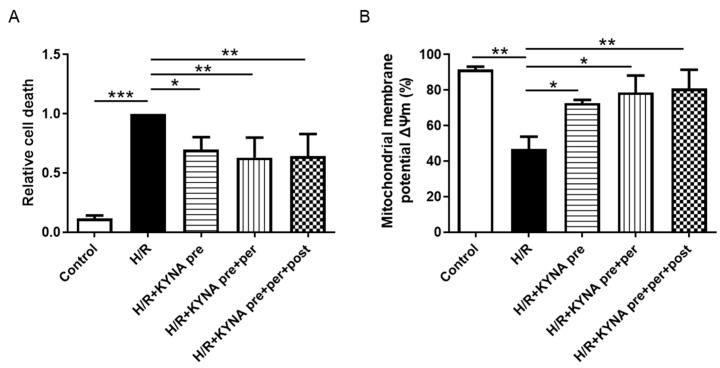
Histograms showing (**A**) relative cell death and (**B**) percentage of cells with conserved mitochondrial membrane potential in control cells that did not undergo hypoxia/reoxygenation (H/R), and cells that underwent H/R, either vehicle (DMSO) or KYNA (1 µM) treated. KYNA treatment was performed either 10 min pre hypoxia in DMEM medium, pre + per hypoxia, or pre + per + post hypoxia for KYNA (n = 6–9 for relative cell death and n = 3–7 for mitochondrial membrane potential). Data are expressed as mean ± SEM; * *p* <0.05, ** *p* < 0.01, *** *p* < 0.001.

To evaluate KYNA’s cytoprotective effects in vitro, H9C2 cells were subjected to 4 h and 50 min of hypoxia, followed by 2 h of reoxygenation with either DMSO or KYNA treatments. As shown in Figure 1A, H/R increased cell death compared to the control (1.00 vs. 0.11 ± 0.02; *p* < 0.001). Relative cell death was significantly reduced in the KYNA-treated groups compared to the H/R group (0.69 ± 0.10, 0.63 ± 0.17, 0.64 ± 0.19 for KYNA pre, KYNA pre + per, KYNA pre + per + post vs. 1.00 in H/R, *p* = 0.024, *p* = 0.006, and *p* = 0.007, respectively) (Figure 1A). H/R led to a significant decrease in the number of cells with a conserved mitochondrial membrane potential (Δ*Ψm*) (46.8 ± 7%) compared to the control (91.5 ± 7%, *p* = 0.003). The KYNA treatment was able to significantly increase the number of cells with a conserved Δ*Ψm* (72.72 ± 1.82%, 78.74 ± 9.50%, 80.97 ± 10.50%, *p* = 0.035, *p* = 0.011, *p* = 0.007 vs. H/R; for KYNA pre, KYNA pre + per, KYNA pre + per + post, respectively) (Figure 1B).

### 2.2. KYNA Reduced Infarct Size In Vivo

**Figure 2:**
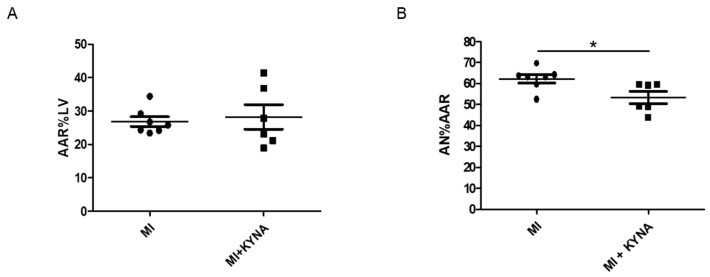
Scatter dot plot showing (**A**) area at risk (AAR) as a percentage of the total left ventricle (LV) and (**B**) area of necrosis (AN) as a percentage of AAR after 2 h of reperfusion (n = 6–7). Data are expressed as mean ± SEM; * *p* < 0.05.

In order to confirm the results obtained in vitro, KYNA or NaOH was injected into male Wistar rats 10 min before a myocardial ischemia/reperfusion. Both the MI and MI + KYNA groups were subjected to 40 min of myocardial ischemia, followed by 2 h of reperfusion. Infarct size was significantly lower in animals receiving KYNA as compared to those receiving the vehicle only (AN%AAR = 53.3 ± 3% in MI + KYNA vs. 62.2 ± 2% in MI, *p* = 0.023), whereas the AAR%LV was similar in the two groups (Figure 2).

### 2.3. KYNA Did Not Influence the Mitochondrial Metabolic Function

**Figure 3:**
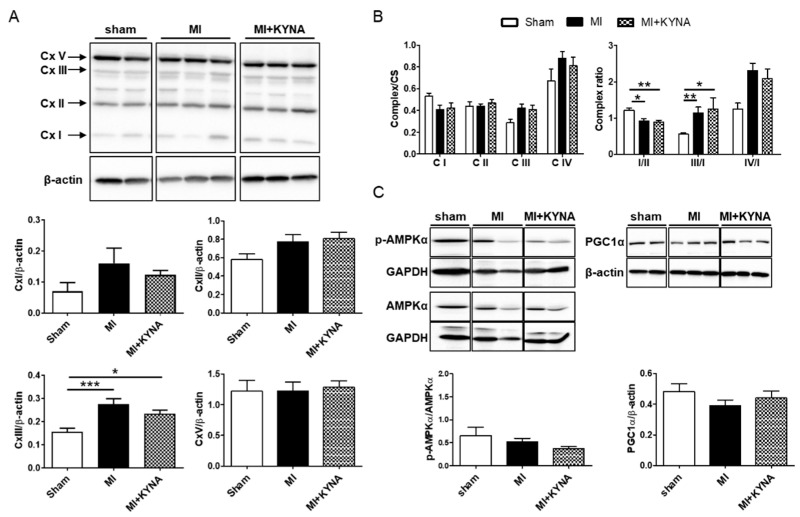
Mitochondrial respiratory chain complex, PGC1α and AMPKα protein expression by means of Western blot and mitochondrial respiratory chain complex activity by spectrophotometry. (**A**) Representative immunoblots and histograms showing quantification of protein expression for the mitochondrial respiratory chain complex in non-ischemic (sham), ischemic + vehicle (MI), and ischemic + kynurenic acid (MI + KYNA) left ventricle (LV) samples after 2 h of reperfusion (n = 5–10). β-actin or GAPDH was used as loading control. (**B**) Histograms showing complex over citrate synthase activity ratio (I/CS, II/CS, III/CS, IV/CS) or complex ratio activity (I/II, III/I, IV/I) in sham, MI, and MI + KYNA LV samples after 2 h of reperfusion (n = 5–10). (**C**) Representative immunoblots and histograms showing quantification of protein expression for AMPKα and PGC1α in sham, MI, and MI + KYNA LV samples after 2 h of reperfusion (n = 5–10). Data are expressed as mean ± SEM; * *p* < 0.05, ** *p* < 0.01, *** *p* < 0.001.

As shown in Figure 3A, complex III proteins’ expression was increased in the MI group compared to the sham (0.28 ± 0.02 in MI vs. 0.16 ± 0.02 in sham for complex III, *p* < 0.001). To ensure consistent mitochondrial content between groups, we assessed the CS activity in the same hearts used for the complex activity studies. The CS specific activity did not differ among the sham, MI, and MI + KYNA groups. Next, we evaluated the specific activities of complexes I, II, III, and IV, normalized to the CS activity. Despite no significant differences among sham, MI, and MI + KYNA groups in each complex’s specific activity, the equilibrium between respiratory complexes (complex ratio) (Figure 3B, right panel) was significantly decreased for complex ratio I/II in the MI and MI + KYNA groups compared to sham group (1.21 ± 0.07 in sham vs. 0.92 ± 0.06 in MI, *p* = 0.02 and vs. 0.88 ± 0.06 in MI + KYNA, *p* = 0.009) and significantly increased for complex ratio III/I in the MI and MI + KYNA groups compared to sham group (0.55 ± 0.04 in sham vs. 1.13 ± 0.17 in MI, *p* = 0.002 and 1.24 ± 0.32 in MI + KYNA, *p* = 0.25). The AMPKα’s, a metabolic sensor regulating PGC1α’s activity, phosphorylation levels were not significantly different among the groups. PGC1α protein expression, a transcription factor mediating mitochondrial biogenesis and oxidative phosphorylation, was not different among the sham, MI, and MI + KYNA groups (Figure 3C).

### 2.4. KYNA Reduced Foxo3α, Akt and ERK1/2 Phosphorylation Levels following Myocardial Ischemia/Reperfusion 

**Figure 4:**
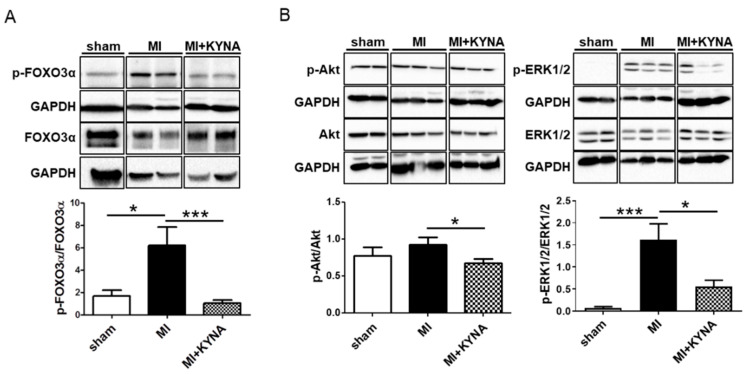
Foxo3α, Akt and ERK1/2 phosphorylation levels by means of Western blot. (**A**) Representative immunoblots and histogram quantification of protein expression levels for p-FOXO3α, FOXO3α and (**B**) p-ERK1/2, ERK1/2, p-Akt, Akt, in non-ischemic (sham), ischemic + vehicle (MI), and ischemic + kynurenic acid (MI + KYNA) left ventricle samples following 15 min of reperfusion (n = 6–9). GAPDH was used as the loading control. Data are expressed as mean ± SEM. * *p* < 0.05, *** *p* < 0.001.

FOXO3α is a transcription factor known to regulate the expression of genes implicated in essential cell functions (including oxidative stress, and mitophagy). After 15 min of reperfusion, FOXO3α phosphorylation levels were significantly increased in the MI group compared to the sham group (6.22 ± 1.61 vs. 1.68 ± 0.51, *p* = 0.044), whereas the KYNA treatment significantly reduced FOXO3α phosphorylation levels (0.89 ± 0.36, *p* < 0.001 vs. MI) (Figure 4A). FOXO3α’s phosphorylation is regulated by Akt and ERK1/2. Hence, we evaluated the potential effect of KYNA on their phosphorylation levels following 15 min of reperfusion. Myocardial ischemia/reperfusion induced an increase in ERK1/2 phosphorylation levels (1.61 ± 0.36 in the MI group vs. 0.07 ± 0.04 in the sham group, *p* < 0.001). The KYNA treatment was associated with a significant decrease in ERK1/2 phosphorylation (0.56 ± 0.14 in MI + KYNA, *p* = 0.024), as well as a significant decrease in Akt phosphorylation compared to the MI group (0.67 ± 0.06 in MI + KYNA vs. 0.92 ± 0.10 in MI, *p* = 0.047) (Figure 4B).

### 2.5. KYNA Stimulated Antioxidant Defense following Myocardial Ischemia/Reperfusion

**Figure 5:**
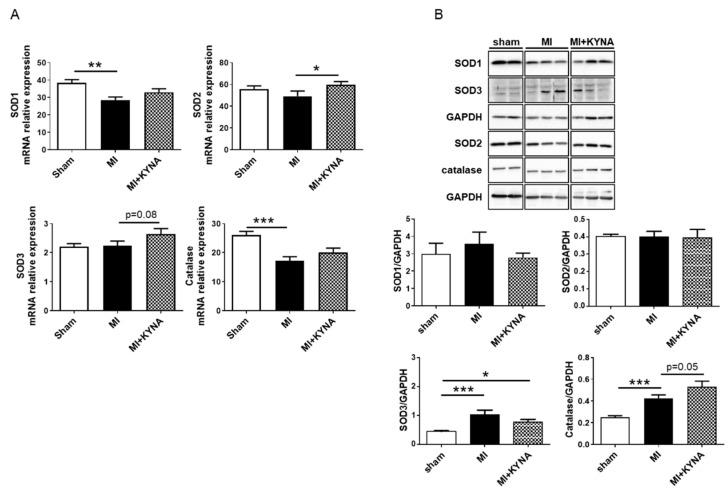
Antioxidant markers mRNA and protein expression, respectively, by means of RT-qPCR and Western blot. (**A**) SOD1, 2, 3, and catalase mRNA expression in non-ischemic (sham), ischemic + vehicle (MI), and ischemic + kynurenic acid (MI + KYNA) left ventricle samples following 2 h of reperfusion (n = 6–11). (**B**) Representative immunoblots and histogram quantification of protein expression for SOD1, 2, 3 and catalase in the sham, MI, and MI + KYNA groups following 2 h of reperfusion (n = 6–9). GAPDH was used as the loading control. Values are expressed as mean ± SEM. * *p* < 0.05, ** *p* < 0.01, *** *p* < 0.001.

KYNA has been reported to exert reactive oxygen species (ROS) scavenger properties. Thus, we evaluated mRNA as well as protein expression of SOD1, SOD2, SOD3, and catalase. The SOD1 and catalase mRNA levels were significantly decreased following MI (28.50 ± 1.81 in the MI vs. 38.33 ± 1.95 in the sham for SOD1, *p* = 0.004; 17.21 ± 1.39 in MI vs. 26.08 ± 1.24 in sham for catalase, *p* < 0.001) whereas SOD2 and SOD3 mRNA remained unchanged. The KYNA treatment induced a significant increase in SOD2 mRNA expression (59.68 ± 2.96 in the MI + KYNA vs. 49.33 ± 4.61 in the MI, *p* = 0.049) and an increase, though not significant, in SOD3 mRNA levels (2.64 ± 0.18 in MI + KYNA vs. 2.24 ± 0.15 in MI, *p* = 0.08) (Figure 5A). The SOD1 and SOD2 protein levels were comparable among all groups. The SOD3 protein levels were significantly higher in the MI (1.02 ± 0.16) vs. the sham group (0.44 ± 0.02; *p* < 0.001). Catalase protein levels were also significantly higher in the MI (0.42 ± 0.03) vs. the sham group (0.25 ± 0.01; *p* = 0.007). Finally, the KYNA treatment exhibited a trend towards increasing catalase protein levels compared to the MI group (0.53 ± 0.05 in MI + KYNA vs. 0.42 ± 0.03 in MI, *p* = 0.055) (Figure 5B).

### 2.6. KYNA Increased Mitophagy Markers following Myocardial Ischemia/Reperfusion

**Figure 6:**
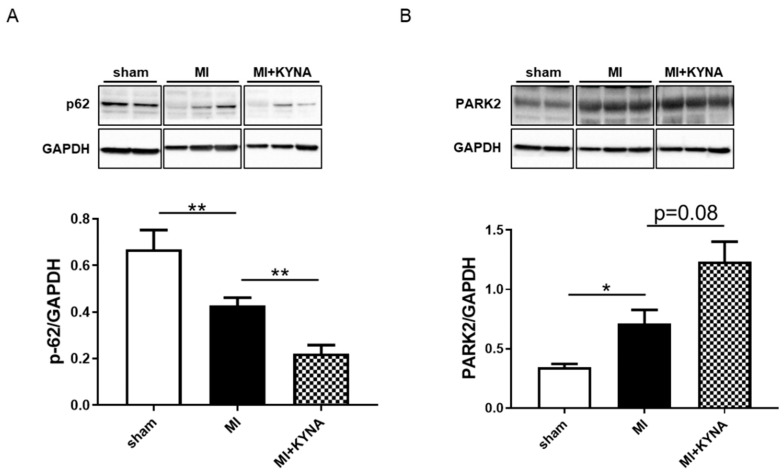
Mitophagy protein markers protein expression levels using Western blot. Representative immunoblots and histogram quantification of protein expression for (**A**) p62 and (**B**) PARK2 in non-ischemic (sham), ischemic + vehicle (MI), and ischemic + kynurenic acid (MI + KYNA) left ventricle samples after 2 h of reperfusion (n = 5–7). GAPDH was used as the loading control. Values are expressed as mean ± SEM. * *p* < 0.05, ** *p* < 0.01.

We assessed whether KYNA was capable of stimulating mitophagy as a cardioprotective mechanism. P62 protein levels were significantly decreased following myocardial ischemia/reperfusion compared to the sham intervention (0.67 ± 0.08 in the MI vs. 0.43 ± 0.03 in the sham, *p* = 0.004), decreasing even further following KYNA treatment (0.22 ± 0.04 in MI + KYNA vs. MI, *p* = 0.008). In addition, PARK2 protein levels were significantly increased in the MI group compared to the sham group (0.34 ± 0.03 in sham vs. 0.71 ± 0.11 in MI, *p* = 0.036). The KYNA treatment tended to increase PARK2 protein levels compared to the MI (1.23 ± 0.17 in MI + KYNA, *p* = 0.08) (Figure 6).

## 3. Discussion

Myocardial infarction is a major cause of death and disability in the world [27]. Reperfusion is indispensable to salvage the ischemic myocardium. However, reperfusion injuries still account for up to 50% of the final infarct size [28]. To date, no cardioprotective strategy has been successfully translated to the clinical arena, which is disappointing. In the current study, we sought to explore several potential cardioprotective pathways using both in vivo and in vitro approaches. We demonstrated KYNA to be capable of reducing ischemia/reperfusion injuries in both an in vitro rat cardiomyoblast H9C2 H/R model and an in vivo myocardial infarction rat model. Moreover, our results suggest that KYNA-induced cardioprotection is likely to be associated with a decrease in FOXO3α degradation which could participate in increased mitophagy and antioxidant defense.

The role of the kynurenine pathway, the major route of tryptophan degradation, in the heart is not clear. The first metabolite generated is kynurenine, which in turn can be metabolized into KYNA. Previous studies have demonstrated the neuroprotective abilities of KYNA. In a neonatal rat cerebral ischemia/reperfusion model (left carotid artery ligation), KYNA was administrated at 300 mg/kg 2 h following cerebral hypoxic-ischemia. This induced a reduction in brain lesions [29]. In another model, a middle cerebral artery occlusion rat model, pretreatment with KYNA, similarly administered at 300 mg/kg, reduced cerebral infarct size [30]. There are few data on KYNA’s ability to protect the heart in the myocardial ischemia/reperfusion setting. We previously reported that increased plasmatic concentrations of kynurenine, KYNA’s precursor, were linked to cardioprotection induced by remote ischemic conditioning [20,22]. KYNA’s protective role was described in a mice model of myocardial ischemia/reperfusion injury [25]. Olenchock et al. generated a mice model with an alpha-ketoglutarate (αKG)-dependent dioxygenase’s (Egln1) somatic gene ablation. EGLN1 senses oxygen and regulates the hypoxia-inducible factor (HIF) transcription, thus coordinating adaptive cellular responses to hypoxia/ischemia [31]. These mice were protected against myocardial ischemia/reperfusion injuries via KYNA levels increase. These authors reported that EGLN1′s down-regulation induced an accumulation of circulating α-ketoglutarate, which is the co-factor for KYNA production. KYNA, in turn, was increased, while mediating cardioprotection against myocardial ischemia/reperfusion injuries. They confirmed their findings by re-injecting KYNA into mice 2 h before and 2 h following myocardial ischemia/reperfusion and shown capable of stimulating cardioprotection [25]. However, the mechanisms and signaling pathways by which KYNA mediates cardioprotection were not explored.

Ischemia/reperfusion injuries are associated with a dysfunction in oxidative phosphorylation [32]. Indeed, complex I and III activities are modulated following ischemia [33,34]. These complexes are key sites generating ROS responsible for at least in part mitochondrial and myocardial damage [35,36]. A reversible blockage of complex I (e.g., by amobarbital) during ischemia was shown to largely avoid reperfusion injury [37,38]. Cardioprotection is partly related to lesser ROS damage, better cytochrome c retention, and preservation of outer membrane integrity [37]. In a previous work, KYNA’s effect on mitochondrial respiration was assessed using oxygraphy [39,40,41]. KYNA could act as an oxidative phosphorylation uncoupler, thus decreasing mitochondrial respiration in mitochondria isolated from rat hearts under basal conditions, i.e., without ischemia/reperfusion. We, thus, tested the hypothesis that KYNA could decrease myocardial ischemia/reperfusion injuries by modulating mitochondrial metabolic function. In our model, KYNA did not influence mitochondrial respiratory chain complex I, II, III, and IV protein expression or activities following ischemia/reperfusion. Moreover, PGC1α is an essential regulator of mitochondrial biogenesis [42], with its activity regulated by AMPKα, a metabolic sensor [43,44]. In our work, the phosphorylation levels of AMPKα, as well as protein expression of PGC1α, were not modified by KYNA treatment, suggesting that, in our model, KYNA-induced cardioprotection is not likely to act through either mitochondrial respiratory chain complex activity modulation or regulation of metabolic signaling.

FOXO3α is a transcription factor known for its role in promoting the transcription of genes implicated in mitophagy and antioxidant defense [45,46]. FOXO3α’s activity is regulated by multiple post-translational modifications. FOXO3α phosphorylation via ERK1/2 and Akt [45] is a signal ensuring its degradation [47,48]. In our work, the decrease in FOXO3α phosphorylation was associated with a decrease in ERK1/2 and Akt phosphorylation levels. In a study by Wang et al., FOXO3α was reportedly associated with increased antioxidant gene transcription, thus decreasing myocardial ischemia/reperfusion injuries [49]. We found a significant increase in SOD2 mRNA levels and high trend towards increased catalase protein expression levels in the KYNA-treated group compared to the vehicle-treated one, indicating possible stimulation of antioxidant defense system. Furthermore, we found a trend for an increase in PARK2 levels, along with a decrease in p62 protein levels suggesting an increase in mitophagy. Several studies have shown FOXO3α’s relevance in regulating parkin-mediated mitophagy [50,51]. Indeed, Mei et al. demonstrated a direct interaction between FOXO3α and (PTEN)-induced putative kinase 1 (PINK1) promoter. PINK1 constitutes an essential factor in the parkin-mediated mitochondrial autophagic pathway. Moreover, mitophagy’s relevance has been highlighted in the cardioprotection setting [52]. For instance, parkin-deficient mice presented a larger infarct size compared to that of the wild type, which was associated with reduced mitophagy and an accumulation of defective mitochondria [53]. Moreover, parkin loss in mice abolished the cardioprotective effect of ischemic preconditioning [54]. Therefore, maintaining the integrity of mitophagy in cardiomyocytes proves to be crucial.

KYNA has been identified as a ligand of the recently de-orphanized G protein-coupled receptor 35 (GPR35) [23]. The emerging relevance of GPR35 in cardiovascular diseases was recently documented [55,56]. Like other G protein-coupled receptors, GPR35 has been shown to modulate signaling pathways that may be implicated in myocardial damage GPR35 was found to be positively regulated in the acute myocardial infarction phase and cultured cardiomyocyte hypoxia cell models (HL-1 and neonatal mice cardiomyocytes) [55]. In GPR35^−/−^ mice, cardioprotection by KYNA administered either 2 or 24 h prior to injury in vivo or 10 or 30 min prior to ischemia/reperfusion in ex vivo hearts was completely abrogated [57]. Moreover, in neonatal cardiomyocytes, KYNA decreased resting oxygen consumption in a GPR35-dependent manner, and after simulated I/R, KYNA decreased mitochondrial reactive oxygen species production and preserved mitochondrial membrane potential which is concordant with the results we obtained in vivo and in vitro [57]. Similar to other G protein-coupled receptors, GPR35 has been suggested to modulate ERK1/2 and Akt phosphorylation levels [58,59]. Indeed, using phospho-ERK1/2 immunoblotting, ligand activation of GPR35 was demonstrated. In these studies, 2-oleoyl lysophosphatidic acid and tyrphostin-51 were recognized as GPR35 agonists in this type of experiment. Using pertussis toxin, which abolishes Gα_i/0_ mediated responses, confirmed ERK1/2 phosphorylation stimulation by GPR35 [60]. Therefore, the exact link between KYNA, GPR35, ERK1/2, and Akt is yet to be established.

Taken together, our present study further indicates KYNA as a potential novel and promising cardioprotective therapeutic agent to reduce myocardial ischemia/reperfusion injuries. We improved our understanding of underlying KYNA-mediated cardioprotection mechanisms by exploring several possible pathways. Future directions would be to translate this novel cardioprotective therapy that targets the kynurenine pathway into a clinical setting, where there is a lack of effective cardioprotective strategies and unmet needs of acute MI patients. The role of KYNA should be more comprehensively investigated to promote its clinical applications.

### Limitations

Our in vitro work demonstrated the cytoprotective effects of KYNA in H9C2 cells, a widely employed immortalized cardiomyoblast cell line. These cells present a phenotype differing from that of adult cardiomyocytes. We confirmed KYNA-induced cardioprotection in an in vivo rat myocardial ischemia/reperfusion model and proposed underlying pathways. In order to explore them, we chose to work with a pretreatment model using high KYNA dosing. GPR35 is a well-documented receptor for KYNA. Its implication in the studied signaling pathways remains unclear. A pharmacological antagonism or genetic inhibition of GPR35 could confirm its implication in the activation of potential downstream effectors such as ERK1/2 and Akt. Thus, further studies are needed to further translate our findings into the clinical arena.

## 4. Materials and Methods

### 4.1. Study Design and Methodology

An experimental study was conducted to explore KYNA-induced cardioprotective pathways using both in vitro hypoxia/reoxygenation and in vivo ischemia/reperfusion models. In vitro, H9C2 cells were subjected to hypoxia/reoxygenation and cell viability as well as the mitochondrial membrane potential were evaluated. In vivo, rats were subjected to a preclinical myocardial ischemia/reperfusion. Left ventricular tissue samples collected at the end of the reperfusion period were used to assess infarct size, mitochondrial respiratory chain complex activity, mRNA expression and protein levels.

### 4.2. In Vitro H9C2 Hypoxia/Reoxygenation

**Figure 7:**
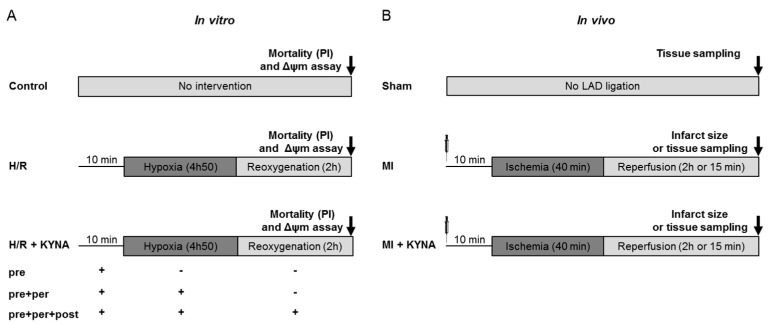
In vitro and in vivo experimental design of protocols and groups. (**A**) In vitro: H9C2 control cells underwent no intervention; cells from the hypoxia/re-oygenation (H/R) groups were submitted to 4 h 50 min of hypoxia followed by 2 h of reoxygenation and were either treated with a vehicle (DMSO) or with kynurenic acid (KYNA 1 µM) 10 min pre hypoxia, pre + per hypoxia, or pre + per + post hypoxia. Cell death quantification as well as mitochondrial membrane potential were performed two hours after reoxygenation. (**B**) In vivo: The sham animals underwent no injection and no left anterior descending coronary artery (LAD) ligation; the MI group underwent 40 min of ischemia followed by 2 h of reperfusion. Then, 10 min before coronary artery occlusion, a vehicle (NaOH 1 M) or KYNA (300 mg/kg) was administrated intraperitoneally. Infarct size assessment using 2,3,5-triphenyltetrazolium chloride (TTC) staining was realized after 2 h of reperfusion; tissue sampling was performed after either 15 min or 2 h of reperfusion.

H9C2-SV40 cells, an immortalized rat cardiomyoblast cell line, were employed for in vitro hypoxia/reoxygenation [61] in order to mimic myocardial ischemia/reperfusion. Cells were cultured in a standard cell culture medium, Dulbecco’s modified eagle medium (DMEM) with high glucose (4.5 g/L), antibiotics (penicillin and streptomycin 10 mL/L), and 10% fetal bovine serum (FBS) (Dutscher, Brumath, France). Hypoxia was induced by washing away the DMEM complete medium three times and replacing it with a glucose- and serum-free isotonic solution (Tyrode’s solution [mM]) (NaCl 130, KCl 5, Hepes 10, MgCl_2_ 1, CaCl_2_ 1.8, pH 7.4). The cells were placed into a hypoxia chamber flushed with a stream of pure nitrogen for 4 h and 50 min at 37 °C. The oxygen rate was kept at 0.5%. Reperfusion was mimicked by replacing Tyrode’s solution with the complete medium in a standard incubator under normoxic conditions for 2 h. The groups were defined as follows (Figure 7A):

– Control group: Cells did not undergo any intervention and were kept in normoxic conditions and a culture medium for 7 h.
– Hypoxia/reoxygenation (H/R) group: Cells underwent H/R with the DMSO (vehicle) treatment 10 min before hypoxia and throughout the procedure.
– H/R + KYNA group: Cells underwent H/R with the KYNA treatment (1 µM) either 10 min before hypoxia (pre), pre + during hypoxia (pre + per), or pre + per + during reoxygenation (pre + per + post).

### 4.3. Cell Death and Mitochondrial Membrane Potential (ΔΨm) Assessment 

At the end of reoxygenation (i.e., 2 h), the cells were detached from the plates using Accutase (PAA Laboratories, Toronto, ON, Canada). Cell death was quantified by flow cytometry (LSR-Fortessa X-20 BD Biosciences, Franklin Lakes, NJ, USA) using 1 µg/mL propidium iodide (PI) (ex: 488 nm; em: 590 nm) (Sigma Aldrich, St. Louis, MO, USA). Cell death in the H/R group was considered equal to one, and other data were expressed as relative mortality compared to H/R. ΔΨm was appreciated using 20 nM DilC1 (1,1′,3,3,3′,3′-hexamethylindodicarbocyanine iodide) (ex: 633 nm; em: 658 nm) (Enzo Life Sciences, Villeurbanne, France), a cationic dye that accumulates in potentiated mitochondria. Results were expressed as the percentage of cells that conserved mitochondria with high ΔΨm. Triplicate samples were prepared for each condition, and a total of 10,000 events were acquired by fluorescence-activated cell sorting (FACS) for each sample [61].

### 4.4. Animal Studies

Male adult Wistar rats, aged 8 to 10 weeks and weighing 250 to 300 g, were used in this study. They were kept in a temperature-controlled room (22 ± 2 °C) with an adequate 12-h light/12 h dark cycle. Food and water were available ad libitum. All experiments were conducted in agreement with the guidelines from EU Directive 2010/63/EU, French Decree no. 2013-118 of the European Parliament on the protection of animals used for scientific purposes. The protocol was approved by the Ethics Committee in Animal Experimentation of Pays de la Loire and by the French Ministry of Higher Education and Research (APAFIS#8668-20 170 12417473589 v3).

Hearts were excised under deep anesthesia (60 mg/kg of sodium pentobarbital (Exagon^®^, Axience, France). Afterwards, in one set of experiments, hearts were excised for infarct size assessment and, in another set of experiments, freeze-clamped and stored at −80 °C for mRNA expression analysis, protein analysis, and assessment of the mitochondrial respiratory chain complex activity.

### 4.5. Myocardial Ischemia/Reperfusion Rat Model

The rats were anesthetized by means of an intraperitoneal injection of 60 mg/kg of sodium pentobarbital (Exagon^®^, Axience, France), orotracheally intubated, and mechanically ventilated with room air by means of a small animal ventilator (SAR-830 A/P, CWE, Ardmore, PA, USA), as previously described [21]. Body core temperature was maintained at 37 ± 0.5 °C (HB101/2 RS; Bioseb, Vitrolles, France). The pericardium was removed to expose the heart after a median sternotomy. Coronary occlusion was induced by carrying out a left anterior descending coronary artery (LAD) ligature. Using a 7.0 monofilament suture (Premio 7.0, Peters Surgical, Boulogne-Billancourt, France) passed through a short length of tubing (PE50), a reversible snare was performed and clamped onto the epicardial surface directly above the coronary artery. The occurrence of epicardial cyanosis and dyskinesia of the ischemic region confirmed ischemia. Following 40 min of occlusion, reperfusion was achieved by loosening the snare and confirmed by observing an epicardial hyperemic response. The anesthesia depth was checked by toe pinch before and during surgery. An extra dose of 30 mg/kg pentobarbital was injected in the event of positive nociceptive response. Rats were randomly assigned to one of the following groups (Figure 7B):

– Sham group: animals undergoing all the surgical procedure except ligature of the coronary artery.
– MI group: animals undergoing myocardial ischemia/reperfusion and injected 10 min before ischemia with NaOH 1 M (vehicle).
– MI + KYNA group: animals undergoing myocardial ischemia/reperfusion and injected 10 min before ischemia with 300 mg/kg KYNA (Sigma Aldrich, St. Louis, MO, USA). The dose was chosen based on previously published data [30,62].

### 4.6. Area at Risk and Infarct Size Determination

Following 120 min of reperfusion, the hearts of the vehicle and KYNA-treated groups were excised, with the LAD re-occluded using the monofilament suture kept in place. The area at risk (AAR) was outlined after a retrograde perfusion with Evans blue (1%). From the apex to the base, the heart was cut into five equal slices, then incubated with a 1% solution of 2.3.5-triphenyltetrazolium chloride (TTC) (Sigma-Aldrich, St. Louis, MO, USA) in phosphate buffer at pH 7.4 and 37 °C. TTC staining enabled the distinction between infarcted myocardium in white and viable myocardium colored brick red. The slices were photographed. Infarct size was quantified using planimetry with Image J software (NIH, Bethesda, MD, USA). The area of necrosis (AN) was expressed as a percentage of the AAR (AN%AAR), and AAR as a percentage of total left ventricular (LV) area (AAR%LV) [63].

### 4.7. Real-Time Quantitative Reverse Transcription Polymerase Chain Reaction (RT-qPCR)

We quantified the expression of genes encoding catalase, and superoxide dismutase 1, 2, 3 (SOD1, 2, 3) following 120 min of reperfusion [64]. Genes encoding hypoxanthine phosphoribosyltransferase (hprt) and glucuronidase beta (gusb) were employed as a reference. Primer sequences have been listed in Table 1.

Total RNA was extracted using the RNeasy MiniKit (Qiagen, Hilden, Germany) according to the manufacturer’s instructions from approximately 30 µg of frozen LV tissue samples from the ischemic zone of the MI and MI + KYNA animals or non-ischemic zone in the sham animals. The cDNA was synthesized using the Quantitect Reverse Transcription Kit (Qiagen, Hilden, Germany) following the manufacturer’s instructions. In a total volume of 20 μL reaction system (10 ng of cDNA), SYBR™ Select Master Mix (Applied Biosystems, Foster City, CA, USA) was applied to perform qPCR using a Lightcycler^®^ 480 II Thermocycler (Roche, Switzerland). The thermal cycling conditions were as follows: 95 °C for 3 min, followed by 40 cycles at 95 °C for 15 s and 60 °C for 1 min. The results of the target genes’ mRNA were normalized on the mean Ct value of the reference genes mRNA and expressed as 2ΔCt, where ΔCt is defined as the difference between the Ct of reference genes and Ct of target genes.

### 4.8. Western Blot (WB) Analysis

The freeze-clamped ischemic (MI and MI + KYNA animals) and non-ischemic (sham) LV rat hearts were employed following 15 min or 120 min of reperfusion for WB analysis as previously described [63]. Briefly, 40 μg of total proteins were separated by SDS-PAGE and transferred into a nitrocellulose or PVDF membrane. The membranes were incubated with antibodies diluted in TBS buffer containing 5% non-fat dried milk, against p-ERK1/2, ERK1/2, p-Akt, Akt, phosphorylated AMP-activated protein kinase (p-AMPKα), AMPKα, p-FOXO3α, FOXO3α (1/1000; Cell Signaling Technology, Danvers, MA, USA), and catalase (1/1000; Sigma-Aldrich, St. Louis, MO, USA). Total OXPHOS Rodent WB Antibody Cocktail (1/250; Abcam, Cambridge, United Kingdom), peroxisome proliferator-activated receptor gamma co-activator 1alpha (PGC1α), SOD2 (1/1000, Abcam, Cambridge, United Kingdom) SOD1 (1/500, Enzo Life Sciences, New York, NY, USA), SOD3, nucleoporin p62 (p62) (1/1000, Enzo Life Sciences, New York, NY, USA), and parkin (PARK2) (1/1000, Abnova, Taipei, Taïwan). GAPDH (1/10,000; Sigma-Aldrich, St. Louis, MO, USA) and β-actin (1/1000; Sigma-Aldrich, St. Louis, MO, USA) were employed as loading controls. The membranes were incubated with appropriate (rabbit or mouse) secondary antibodies (1/5000, Thermo Fisher Scientific, Waltham, MA, USA) conjugated to horseradish peroxidase. The blots were developed using the enhanced chemi-luminescence method. The band densities were analyzed using Image Lab (BioRad, Hercules, CA, USA).

### 4.9. Mitochondrial Respiratory Chain Complex Enzymatic Activity Assessment

The activities of lactate dehydrogenase (LDH), citrate synthase (CS), and the electron transport chain complexes (complexes I–IV) were spectrophotometrically measured at 37 °C with a UV spectrophotometer (SAFAS, UVmc2, Monaco) in the sham, MI, and MI + KYNA LV muscle homogenates following 2 h of reperfusion [65,66]. Homogenates were obtained after repeating the homogenization and centrifugation step twice (20 min at 650× *g*) while discarding the pellet.

The CS activity was measured in a reaction medium consisting of 100 mM Tris·HCl pH 8.1, 150 µM 5.5′-dithio-bis (2-nitrobenzoic acid) (DTNB), 50 µM oxaloacetate, 30 µM acetyl-CoA, and 0,1% Triton X-100. After 2 min of incubation, the reaction was initiated by adding 10 µL/mL homogenate, with the change in optical density at 412 nm recorded over 1 min.

NADH ubiquinone reductase (complex I) activity was assayed in KH_2_PO_4_ buffer pH 7.5, 3.75 mg/mL bovine serum albumin (BSA), 100 µM decylubiquinone, and 10 µL/mL homogenate, with (to determine background rates, subsequently subtracted) or without 10 µM rotenone. After 2 min of incubation at 37 °C, the reaction was initiated by adding 0.1 mM NADH. The activity was measured at 340 nm by monitoring NADH oxidation over 2 min.

The succinate dehydrogenase (complex II) activity was measured after the reduction of 2,6-dichlorophenolindophenol (DCPIP) at 600 nm in a buffer containing 50 mM KH_2_PO_4_, 2.5 mg/mL BSA, 6.5 µM rotenone, 5 µmg/mL antimycin, 25 mM succinate, 1 mM KCN, and 100 μM DCPIP, pH 7.5. After 2 min of incubation at 37 °C with 15 µL of homogenate, the reaction was initiated by adding 100 μM decylubiquinone, with the optical density recorded for 2 min.

The ubiquinone-cytochrome c reductase (complex III) activity was determined by monitoring the reduction of cytochrome c at 550 nm. Overall, 10 µL/mL homogenate was incubated for 60 s in a reaction medium consisting of 100 mM KH_2_PO_4_ pH 7.5, 250 µM ethylenediaminetetraacetic acid (EDTA), 1 mg/mL BSA, 1 mM KCN, and 100 μM oxidized cytochrome c, with or without 5 µg/mL antimycin (non-enzymatic reduction of cytochrome c). The reaction was initiated by adding 100 μM decylubiquinol, and the optical density was measured over 40 s. The specific complex III activity was calculated by subtracting the activity of the non-enzymatic reaction from that of the total activity.

The cytochrome-c oxidase (complex IV) activity was measured by monitoring the oxidation of reduced cytochrome c at 550 nm. An 80 μM solution of reduced cytochrome c (92–97% reduced using dithionite) in 55 mM KH_2_PO_4_, pH 7.0 was pre-incubated over 2 min. The reaction was initiated by adding 10 µL/mL of homogenate, with the change in optical density measured over 40 s.

A control with beef heart mitochondria was executed in parallel with each set of samples to ensure proper running of the experiments. The cellular protein content was determined using the BCA protein assay kit (Thermo Scientific, Waltham, MA, USA) with BSA as standard. Specific complex activities were expressed as a ratio of and normalized to CS activity.

### 4.10. Statistical Analysis

Statistical analysis was performed using SPSS Statistics v.17.0 software (SPSS Inc., Chicago, IL, USA). After verifying data’s distribution’s normality using Shapiro–Wilk test, the appropriate statistical test was performed. Differences between the two groups (for AAR%LV and AN%AAR) were evaluated using Student’s *t*-test. For multiple group comparison, either a one-way ANOVA followed by an LSD post hoc test was performed or a Kruskal–Wallis test followed by a Pairwise Comparison’s post hoc test. Data are expressed as mean ± standard error of the mean (SEM); a *p* < 0.05 was considered statistically significant.

## 5. Conclusions

KYNA reduced ischemia/reperfusion injuries in both in vitro and in vivo models. KYNA-mediated cardioprotection was associated with decreased ERK1/2 and Akt phosphorylation levels, which may have contributed to a decrease in FOXO3α phosphorylation, possibly linked with increased mitophagy and antioxidant defense. Additional studies are needed to establish the exact link between these different downstream effectors. For instance, inhibition of underlying signaling pathways would validate their implication in the conferred cardioprotection. Moreover, GPR35′s implication would require confirmation by testing KYNA’s cardioprotective effects in GPR35 knockout mouse model or using antagonists of GPR35. Further in-depth investigations of KYNA’s cardioprotective mechanisms using other rodents and mammalian models are required. Thus, supplementation of KYNA might be helpful to improve acute myocardial infarction management. 

## Figures and Tables

**Table 1:** Forward and reverse primer sequences for target and reference genes.

Gene	NCBI Genbank	Forward Sequence	Reverse Sequence
*cat*	*NM_012520.2*	5′-ttgccaaccacctgaaagat-3′	5′-agggtggacgtcagtgaaat-3′
*gusb*	*NM_017015.2*	5′-ctctggtggccttacctgat-3′	5′-cagactcaggtgttgtcatcg-3′
*hprt*	*NM_012583.2*	5′-gaccggttctgtcatgtcg-3′	5′-acctggttcatcatcactaatcac-3′
*sod1*	*NM_017050.1*	5′-ggtccagcggatgaagag-3′	5′-ggacacattggccacacc-3′
*sod2*	*NM_017051.2*	5′-attgccgcctgctctaatc-3′	5′-gatagtaagcgtgctcccaca-3′
*sod3*	*NM_012880.1*	5′-cttgggagagcttgtcaggt-3′	5′-caccagtagcaggttgcaga-3′

## Data Availability

Data is contained within the article.
